# Intraoperative Volume Decay Measured Through Mechanical Ventilation as a Predictor of Prolonged Postoperative Air Leak After Uniportal Video-Assisted Thoracic Surgery Lung Resection

**DOI:** 10.3390/jpm16080434

**Published:** 2026-08-17

**Authors:** Michele Salati, Alberto Roncon, Gian Marco Guiducci, Michela Tiberi, Mara Romito, Anna Chiara Nanto, Francesco Xiumè, Rosanna Coltrinari, Stefano Falcetta, Paolo Gentili, Abele Donati, Majed Refai

**Affiliations:** 1 Thoracic Surgery Unit, University Hospital of Marche, Via Conca 71, 60126 Ancona, Italy; alberto.roncon@ospedaliriuniti.marche.it (A.R.); gianmarco.guiducci@ospedaliriuniti.marche.it (G.M.G.); michela.tiberi@ospedaliriuniti.marche.it (M.T.); mara.romito@ospedaliriuniti.marche.it (M.R.); annachiara.nanto@ospedaliriuniti.marche.it (A.C.N.); francesco.xiume@ospedaliriuniti.marche.it (F.X.); majed.refai@ospedaliriuniti.marche.it (M.R.); 2 Anesthesia and Intensive Care Clinic, Polytechnic University of Marche, Via Tronto 10/a, 60126 Ancona, Italy; rosanna.coltrinari@ospedaliriuniti.marche.it (R.C.); stefano.falcetta@ospedaliriunit.marche.it (S.F.); paolo.gentili@ospedaliriuniti.marche.it (P.G.); abele.donati@ospedaliriuniti.marche.it (A.D.)

**Keywords:** prolonged air-leak, video assisted thoracic surgery, VATS, volume decay, mechanical ventilation, intraoperative monitoring, lung resection, personalized surgery, thoracic surgery, postoperative complications

## Abstract

**Background/Objectives**: Prolonged postoperative air leak (PAL) remains one of the most common complications after lung resection, significantly affecting postoperative recovery and healthcare utilization. While several preoperative risk factors have been identified, reliable intraoperative predictors are still lacking. This study aimed to evaluate whether intraoperative ventilator-derived parameters—volume decay (Vol-Decay) and pressure decay (Pres-Decay)—are associated with the development of PAL following uniportal video-assisted thoracic surgery (VATS) lung resection. **Methods**: We conducted a single-center study including 277 consecutive patients undergoing uniportal VATS lung resection for neoplastic disease between May 2023 and April 2024. At the end of the surgical procedure, intraoperative Vol-Decay and Pres-Decay were measured using the mechanical ventilator under standardized conditions. PAL was defined as an air leak persisting for more than 5 days within 30 days after surgery. Univariate and multivariate analyses were performed to identify independent predictors of PAL, and the optimal Vol-Decay cut-off was determined using Youden’s index. **Results**: PAL occurred in 18 patients (6.5%). At univariate analysis, higher smoking exposure, lower FEV1, lower FEV1/FVC ratio, and higher Vol-Decay were significantly associated with PAL. At multivariable analysis, only FEV1 (*p* = 0.013) and Vol-Decay (*p* = 0.038) remained independent predictors. The optimal Vol-Decay cut-off was 22 mL (AUC 0.64), with high specificity (0.94) but low sensitivity (0.34). Patients with Vol-Decay > 22 mL showed a significantly higher incidence of PAL compared to those with lower values (27.3% vs. 4.7%). **Conclusions**: Intraoperative Vol-Decay represents a simple, objective, and reproducible parameter associated with the development of prolonged air leak after uniportal VATS lung resection. Its measurement may enable real-time risk stratification and support personalized intraoperative decision-making, potentially guiding targeted corrective strategies. Further prospective multicenter studies are warranted to validate these findings and confirm their clinical applicability.

## 1. Introduction

Postoperative prolonged air leak (PAL), defined as a persistent peripheral air leak through the visceral pleura lasting more than 5 days [1], is one of the most common complications following lung resection. It affects approximately 10% of patients undergoing anatomic resections and 5% of those undergoing wedge resections [2] and may contribute to the development of additional respiratory and infectious complications.

Although numerous studies have investigated risk factors and predictive models for PAL after lung resection, only a limited number have focused on intraoperative parameters as potential predictors [3,4,5,6,7]. The identification of reliable intraoperative predictors would allow surgeons to implement corrective strategies during the procedure, potentially reducing the risk of persistent postoperative air leak.

Traditionally, air leaks are assessed at the end of open surgery by submerging the lung in saline and observing for air bubbles [8]. However, this method is technically challenging in video-assisted thoracic surgery (VATS) [9]. Therefore, the identification of objective intraoperative parameters capable of predicting PAL during VATS procedures would be highly desirable, enabling timely corrective interventions.

Experimental models have demonstrated a clear relationship between pleural injury, airflow through peripheral broncho-pleural fistulas, and airway pressure [10,11]. In an ideal closed system consisting of the mechanical ventilator and the lungs, inspiratory and expiratory volumes should be equal in the absence of leaks. Similarly, during an inspiratory pause, airway pressure should remain stable unless air leakage is present.

Based on these principles, the aim of the present study was to evaluate whether intraoperative volume decay (Vol-Decay)—defined as the difference between inspiratory and expiratory volumes—and pressure decay (Pres-Decay) within the lung–ventilator system are associated with the development of prolonged postoperative air leak following uniportal VATS lung resection.

## 2. Materials and Methods

We conducted a study including patients who underwent lung resection for neoplastic disease at our institution between May 2023 and April 2024.

All patients were evaluated by a multidisciplinary team including thoracic surgeons, oncologists, pulmonologists, radiologists, and pathologists, who confirmed the indication for surgical treatment.

Preoperative functional assessment was performed according to international guidelines [12]. Contraindications to anatomic lung resection included unstable cardiac disease, predicted postoperative forced expiratory volume in 1 s (ppoFEV1) and/or predicted postoperative diffusing capacity for carbon monoxide (ppoDLCO) < 30%, in association with a peak oxygen consumption (VO_2_ peak) < 10 mL/kg/min. Both ppoFEV1 and ppoDLCO were estimated based on the number of functional lung segments to be resected, as determined by bronchoscopy and computed tomography (CT), or ventilation/perfusion scintigraphy when required.

All procedures were performed using a uniportal VATS approach, as previously described [13,14]. Briefly, the technique involves a single 3–4 cm incision at the 5th intercostal space along the anterior axillary line, through which the thoracoscope and surgical instruments are introduced. Both anatomic and non-anatomic resections, with lymph node dissection when indicated, were performed following systematic surgical steps [15].

Standardized perioperative management protocols were applied in all patients, according to our institutional Enhanced Pathway of Care (EPC), previously reported [13,16].

The study was notified to the Ethics Committee of the Marche Region and conducted in accordance with the Declaration of Helsinki and applicable national regulations. Study-specific informed consent was formally waived through the institutional Data Protection Impact Assessment (DPIA) procedure, in accordance with the applicable regulatory framework governing the use of anonymized patient data for research purposes.

### 2.1. Intraoperative Volume Decay and Pressure Decay Measurement

At the end of the surgical procedure, after chest tube placement through the uniportal incision and restoration of double-lung ventilation, intraoperative measurements of Volume Decay (Vol-Decay) and Pressure Decay (Pres-Decay) were performed prior to wound closure.

Vol-Decay was assessed under standardized protective ventilation settings (tidal volume 8 mL/kg, respiratory rate 12 breaths/min, and positive end-expiratory pressure of 5 cmH_2_O). For each respiratory cycle, inspiratory and expiratory volumes (mL) were recorded. The difference between inspiratory and expiratory volumes represented the decay for a single cycle. Vol-Decay was defined as the mean value calculated over 10 consecutive respiratory cycles. Vol-Decay was calculated as the difference between inspiratory and expiratory tidal volumes directly recorded by the mechanical ventilator under a standardized ventilation protocol, thereby minimizing operator-dependent variability.

Pres-Decay was assessed at the beginning of a new respiratory cycle. A controlled inflation maneuver was performed to reach the previously recorded plateau pressure. At the end of inspiration, the lung–ventilator system was maintained closed for 30 s at a PEEP of 5 cmH_2_O. The difference between the plateau pressure and the pressure measured after 30 s represented Pres-Decay.

For each patient, both Vol-Decay and Pres-Decay values were recorded.

### 2.2. Study Design and Statistical Analysis

For the purposes of this study, prolonged air leak (PAL) was defined as an air leak occurring within 30 days after surgery and persisting for more than 5 days.

Air leak was assessed three times daily (8 AM, 1 PM, and 5 PM) using a digital drainage system capable of measuring airflow (mL/min) and intrapleural pressure (cmH_2_O). Chest tubes were maintained under continuous suction at −2 cmH_2_O while in place. Air leak resolution was defined as airflow < 20 mL/min sustained for at least 12 consecutive hours [17]. Chest tube removal was performed from postoperative day 2 onward once this condition was met.

Patients with air leak persisting beyond postoperative day 3 were discharged on postoperative day 4 with a chest tube connected to a Heimlich valve when possible. Follow-up assessments were performed three times per week in the outpatient clinic, evaluating bubbling during coughing after submerging the chest tube in water.

The following preoperative and intraoperative variables were analyzed for association with PAL: sex, age, body mass index (BMI), American Society of Anesthesiologists (ASA) score, smoking exposure (pack-years), forced expiratory volume in 1 s (FEV1), forced vital capacity (FVC), FEV1/FVC ratio, diffusing capacity for carbon monoxide (DLCO), chronic obstructive pulmonary disease (COPD), type of resection, anatomic versus non-anatomic resection, Vol-Decay, and Pres-Decay. Their standardized definition was reported in a previous study [1].

Data were retrieved from a prospectively maintained institutional database. Variables with completeness < 90% and patients lacking primary outcome data were excluded. Missing data ≤ 10% were handled using appropriate imputation methods.

Normality was assessed using the Shapiro–Wilk test. Continuous variables were analyzed using Student’s *t*-test or the Mann–Whitney U test, as appropriate. Categorical variables were analyzed using the chi-square test.

The univariate analysis tested the possible association of the above mentioned preoperative and operative characteristics of the patients with postoperative PAL. Variables with *p* ≤ 0.1 in univariate analysis were included in a stepwise logistic regression model to identify independent predictors of PAL. The optimal Vol-Decay cut-off was determined using Youden’s index [18]. Given the limited number of PAL events, the multivariable model was intentionally kept parsimonious to reduce the risk of overfitting.

Statistical analyses were performed using Stata version 14.0 (StataCorp, College Station, TX, USA).

## 3. Results

A total of 277 consecutive patients undergoing lung resection for neoplastic disease were included. Among these, 173 patients (62.5%) underwent anatomic resections (139 lobectomies/bilobectomies and 34 segmentectomies), while 104 (37.5%) underwent wedge resections.

Baseline characteristics are summarized in Table 1. The mean intraoperative Vol-Decay was 10.8 mL (SD 13.6), and the mean Pres-Decay was 0.7 cmH_2_O (SD 1.7).

Overall, 18 patients (6.5%) developed PAL during the postoperative period.

Univariate analysis (Table 2) showed that patients with PAL had significantly greater smoking exposure (43.2 vs. 26.5 pack-years, *p* = 0.04), lower FEV1 (80.3% vs. 93.8%, *p* = 0.007), and lower FEV1/FVC ratio (0.67 vs. 0.74, *p* = 0.01). In addition, Vol-Decay was significantly higher in patients with PAL compared to those without (19.2 vs. 10.2 mL, *p* = 0.007). Similarly, the type of pulmonary resection (lobectomy, bilobectomy, segmentectomy, or wedge resection) was not significantly associated with the occurrence of prolonged postoperative air leak (*p* = 0.07).

No significant difference in Pres-Decay was observed between groups (1.05 vs. 0.7 cmH_2_O, *p* = 0.4).

In the final multivariable logistic regression model, only FEV1 (OR 0.97, 95% CI 0.95–0.99, *p* = 0.013) and Vol-Decay (OR 1.02, 95% CI 1.00–1.05, *p* = 0.038) remained independent predictors of PAL.

**Figure 1:**
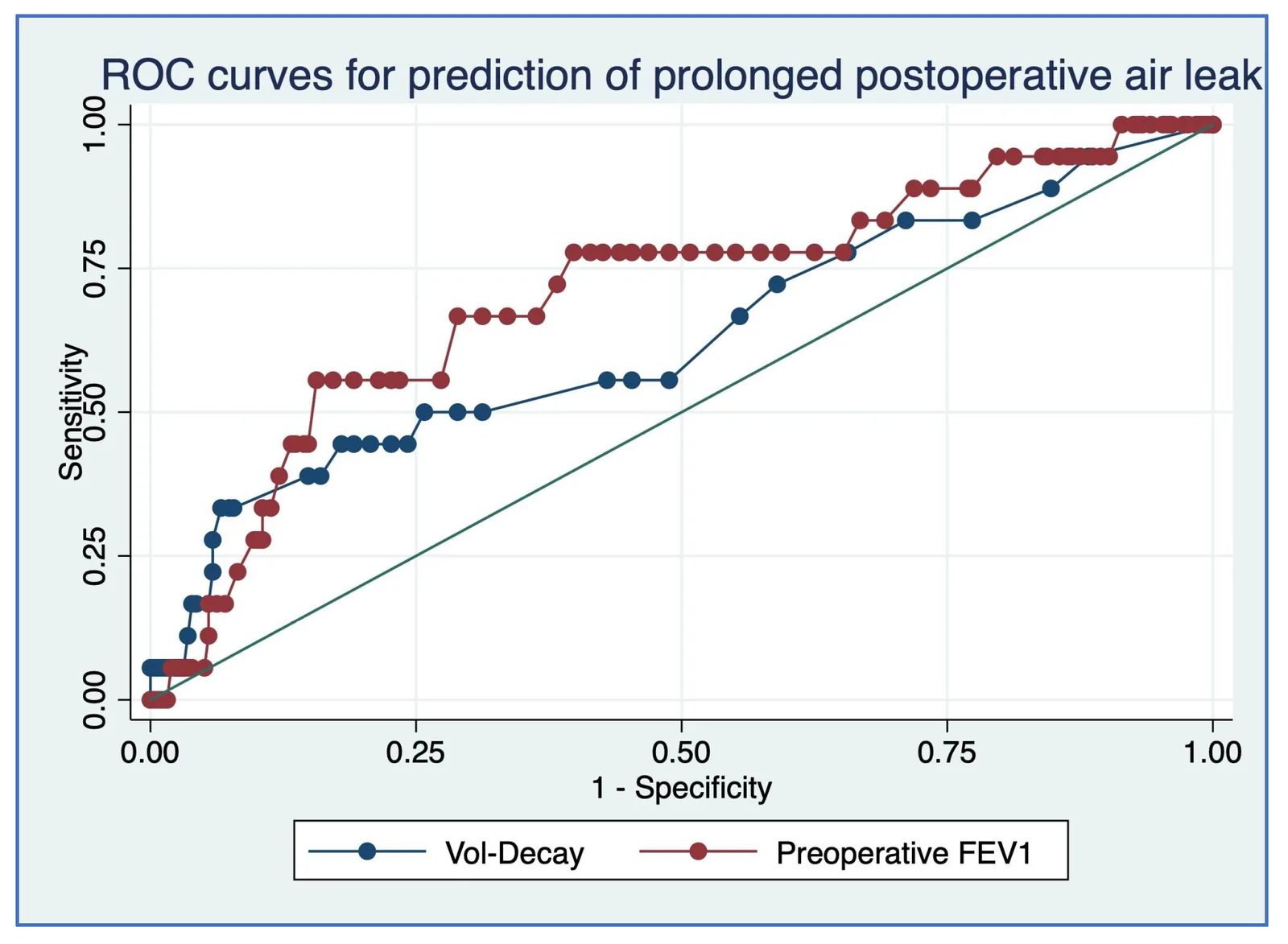
Receiver operating characteristic (ROC) curves for Vol-Decay and preoperative FEV1 in predicting prolonged postoperative air leak. The area under the curve (AUC) was 0.63 (95% CI: 0.47–0.78) for Vol-Decay and 0.70 (95% CI: 0.57–0.84) for preoperative FEV_1_.

The optimal Vol-Decay cut-off value identified using Youden’s index was 22 mL (AUC 0.64, specificity 0.94, sensitivity 0.34). The ROC curves for Vol-Decay and preoperative FEV1 are shown in Figure 1.

**Figure 2:**
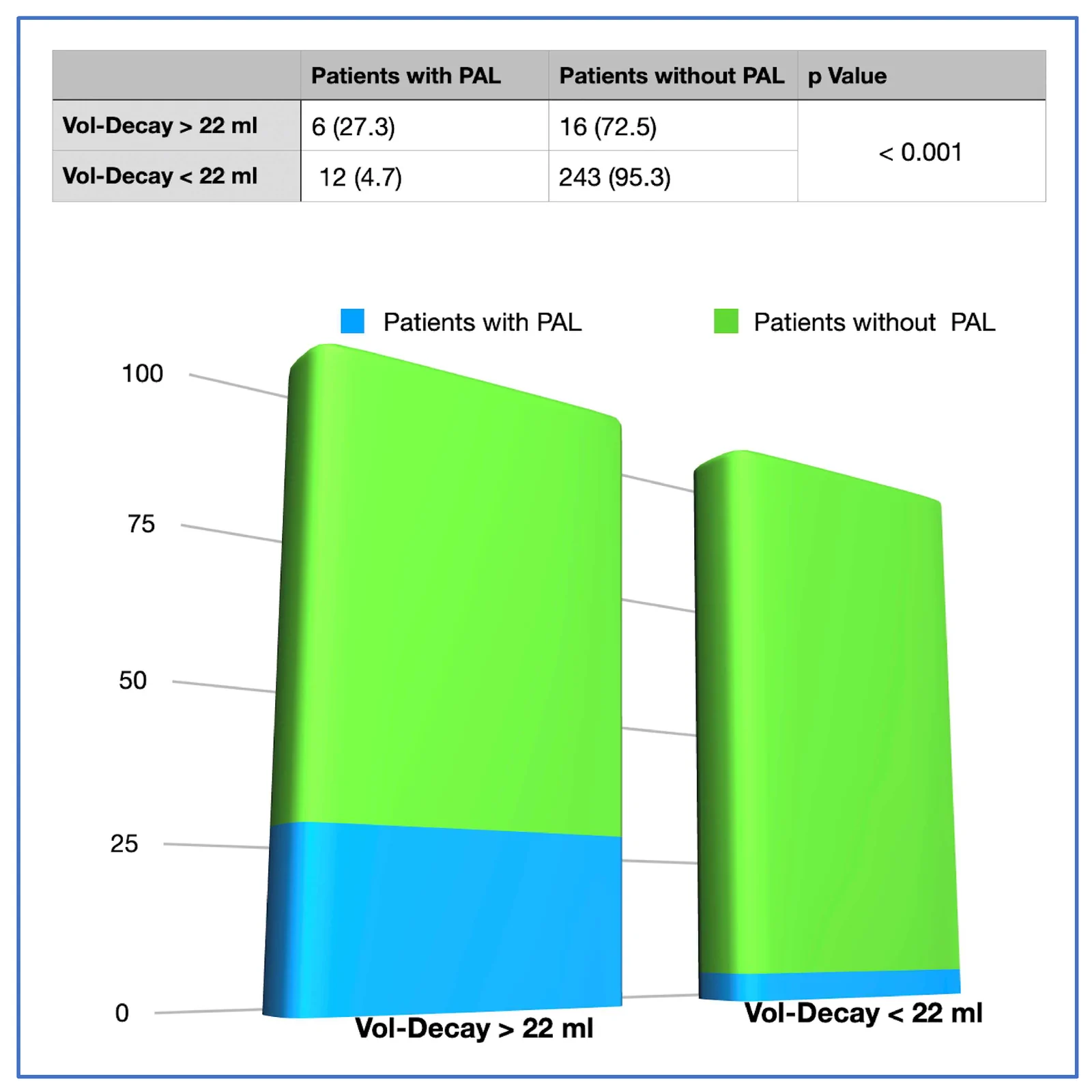
Prolonged air-leak distribution using the threshold of 22 mL volume decay for categorizing the population. Incidence of prolonged postoperative air leak (PAL) according to the optimal Vol-Decay threshold identified by ROC curve analysis. Among patients with Vol-Decay ≥ 22 mL (n = 22), PAL occurred in 6 cases (27.3%), whereas among those with Vol-Decay < 22 mL (n = 255), PAL occurred in 12 cases (4.7%).

Patients with Vol-Decay > 22 mL had a markedly higher incidence of PAL compared to those with lower values (27.3% vs. 4.7%), corresponding to more than a fivefold increase in risk (Figure 2).

## 4. Discussion

The present study aimed to evaluate whether intraoperative ventilator-derived parameters—specifically Volume Decay (Vol-Decay) and Pressure Decay (Pres-Decay)—could predict the occurrence of PAL following uniportal VATS lung resection. The main finding of this analysis is that Vol-Decay represents an independent predictor of PAL, whereas Pres-Decay does not show a significant association.

The overall incidence of PAL in our cohort (6.5%) is consistent with contemporary series, which report rates ranging between 5% and 10% depending on patient characteristics and type of resection. Recent analyses have highlighted not only the variability in PAL incidence but also the heterogeneity in its prevention and management strategies across institutions [7,19]. Despite the widespread adoption of minimally invasive approaches and technological advancements, PAL remains one of the most frequent and clinically relevant complications after lung resection.

The clinical burden of PAL is well established. As originally described by Cerfolio et al., persistent air leak is associated with prolonged chest tube duration, which in turn negatively impacts postoperative recovery [20]. Subsequent studies have further clarified how prolonged drainage contributes to increased postoperative pain and reduced patient compliance with physiotherapy and early mobilization [21,22]. Moreover, PAL has been linked to higher rates of pulmonary and pleural infections, increased readmissions, and delayed hospital discharge [23], ultimately translating into higher healthcare costs and delayed return to normal activities [24].

Given these implications, considerable effort has been devoted to reducing PAL through both preventive and intraoperative strategies. Technical refinements such as fissureless lobectomy and the use of reinforced staplers in fragile or emphysematous parenchyma have shown promising results in lowering the incidence of postoperative air leak [25,26]. In our series, these strategies were routinely applied within a standardized uniportal VATS approach; nevertheless, a non-negligible rate of PAL persisted. This finding underscores a critical point: even optimal surgical technique alone may not be sufficient to eliminate PAL risk, highlighting the need for reliable intraoperative predictive tools.

In this context, the identification of Vol-Decay as an independent predictor of PAL represents a clinically relevant advancement. Unlike preoperative risk factors, which only allow risk stratification before surgery, intraoperative parameters offer the unique opportunity to guide real-time decision-making. Specifically, the detection of increased Vol-Decay at the end of the procedure may prompt the surgeon to perform additional corrective maneuvers, such as targeted suturing, further parenchymal resection, or the application of sealants. An important consideration is whether the predictive value of Vol-Decay simply reflects the extent of pulmonary resection, as a larger resection surface could theoretically generate greater intraoperative air leakage. In our study, however, the type of pulmonary resection was not significantly associated with PAL at univariable analysis, and an additional analysis performed in response to this concern showed that Vol-Decay did not differ significantly across the different types of pulmonary resection (one-way ANOVA, *p* = 0.715). Although these findings do not exclude a contribution of surgical complexity, they suggest that the association between Vol-Decay and PAL is unlikely to be explained solely by the extent of resection. Rather, Vol-Decay may reflect the overall integrity of the residual lung–ventilator system, integrating both patient-related and surgery-related factors. Larger prospective studies are warranted to further clarify the relative contribution of these mechanisms.

From a pathophysiological perspective, our findings are in line with experimental evidence. Servais et al., using an intact murine lung model, demonstrated that both pressure decay and discrepancies between inspiratory and expiratory volumes within a closed lung–ventilator system are proportional to the extent of pleural injury [11]. In our clinical setting, we were able to confirm this relationship only partially, as Vol-Decay—but not Pres-Decay—was significantly associated with clinically relevant postoperative air leak. This discrepancy is likely attributable to fundamental differences between experimental conditions and real-world clinical practice. Specifically, experimental models typically employ standardized and often supraphysiological airway pressures to amplify signal detection, whereas in our study pressure measurements were obtained under physiological conditions using patient-specific plateau pressures, without any modification of routine ventilatory settings. While this approach enhances clinical applicability and safety, it may inherently reduce the sensitivity of Pres-Decay in detecting subtle or low-grade air leaks. Furthermore, the standardized 30 s end-inspiratory occlusion period and ventilator settings adopted in the present study were selected to ensure feasibility and safety within routine clinical practice rather than to maximize diagnostic performance. It is therefore possible that alternative acquisition protocols, including different occlusion durations or ventilatory settings, may improve the ability of Pres-Decay to detect clinically relevant air leaks while preserving its potential applicability in the operating room. Future studies should specifically investigate these aspects.

Importantly, we identified a Vol-Decay threshold of 22 mL as the optimal cut-off for predicting PAL. While the sensitivity of this threshold is limited, its high specificity makes it a potentially valuable rule-in criterion for identifying high-risk patients. Importantly, we identified a Vol-Decay threshold of 22 mL as the optimal cut-off for predicting PAL. Although the overall discriminative performance of Vol-Decay was only moderate (AUC 0.64), this finding should be interpreted within the clinical context in which the parameter is intended to be used. Rather than serving as a standalone predictor, Vol-Decay may represent an adjunctive intraoperative marker that provides additional objective information beyond established preoperative risk factors by providing objective information on the mechanical integrity of the lung–ventilator system at the end of resection. Moreover, while the identified threshold showed limited sensitivity, its high specificity may help identify a subgroup of patients at particularly high risk of PAL who could benefit from additional intraoperative corrective measures, such as targeted suturing, reinforcement of staple lines, or the application of sealants. Alternative cut-off values could be selected to prioritize sensitivity over specificity. For example, lowering the threshold from 22 mL to 18 mL would increase sensitivity from 33.3% to 44.4%, while reducing specificity from 92.7% to 82.2%. The selected 22 mL threshold was therefore intended to maximize specificity, identifying a subgroup of patients at particularly high risk of PAL who may benefit most from additional intraoperative preventive measures. Notably, patients with a Vol-Decay greater than 22 mL experienced more than a fivefold higher incidence of PAL compared with those below the threshold.

Consistent with previous literature, reduced preoperative FEV1 also emerged as an independent predictor of PAL [27]. However, while FEV1 reflects baseline lung function and tissue fragility, it does not account for intraoperative factors or the actual mechanical integrity of the remaining lung. In contrast, Vol-Decay provides a direct functional assessment of the lung–ventilator system at the end of resection, potentially capturing both pre-existing and surgery-induced vulnerabilities. For this reason, these parameters should be viewed as providing complementary clinical information rather than alternative measures of the same physiological process. While FEV1 reflects baseline pulmonary function before surgery, Vol-Decay provides an intraoperative assessment of the mechanical integrity of the residual lung–ventilator system following resection.

From a clinical perspective, Vol-Decay should not be interpreted as a standalone diagnostic tool but rather as an objective intraoperative parameter that may complement previously established clinical and surgical risk factors. Although its discriminative ability was only moderate and the proposed threshold requires external validation, intraoperative identification of increased Vol-Decay may help surgeons recognize patients at higher risk of PAL and prompt additional corrective measures, such as targeted suturing, further parenchymal resection, or the selective use of sealants when deemed appropriate. Future multicenter prospective studies are warranted to validate these findings and determine whether Vol-Decay-guided intraoperative strategies can improve postoperative outcomes.

Several limitations of this study should be acknowledged. First, although the multivariable model was intentionally kept parsimonious, the final model included two independent predictors, resulting in an events-per-variable ratio of 9 (18 events for 2 variables). Although close to the commonly recommended threshold, this relatively limited ratio may have reduced statistical power and increased the risk of overfitting and instability of the regression coefficients. In addition, several established risk factors for prolonged postoperative air leak, including the severity of pulmonary emphysema, fissure completeness, and the intraoperative use of sealants, were not systematically recorded and therefore could not be included in the multivariable analysis. Their potential confounding effect cannot be excluded and should be addressed in future prospective studies specifically designed to integrate these variables into predictive models. Consequently, the identified independent predictors should be interpreted with appropriate caution and considered hypothesis-generating until confirmed in larger prospective studies. Second, the use of standard clinical ventilators introduces potential variability in volume and pressure measurements, as previously reported in bench studies [28]. Third, although digital drainage systems were used, some degree of variability in postoperative air leak assessment cannot be entirely excluded. Fourth, the single-center design and the adoption of specific surgical and perioperative protocols may limit the generalizability of our findings. Although Vol-Decay was measured using a standardized intraoperative protocol, differences in ventilator equipment, ventilatory settings, and institutional perioperative management across centers may influence the measured values. In addition, variations in postoperative chest drainage management may affect the clinical identification of PAL, potentially limiting the applicability of the proposed 22 mL threshold across different institutions. Therefore, the proposed cut-off should be considered hypothesis-generating until externally validated in independent patient cohorts. Further prospective multicenter studies are needed to establish its reproducibility, predictive performance, and generalizability across different clinical settings. Finally, although Vol-Decay was derived from objective ventilator-generated measurements acquired under a standardized protocol, formal assessment of measurement reproducibility and interobserver variability was not performed. Despite these limitations, this study provides novel evidence supporting the role of intraoperative ventilator-derived parameters in predicting PAL. Future prospective, multicenter studies are warranted to validate these findings and to explore whether Vol-Decay–guided intraoperative strategies can effectively reduce the incidence of prolonged air leak.

## 5. Conclusions

Intraoperative Vol-Decay measured through the mechanical ventilator represents a simple and objective parameter associated with the development of prolonged air leak after uniportal VATS lung resection.

A threshold value of 22 mL may help identify patients at higher risk, and could complement intraoperative clinical assessment when considering additional preventive measures. Further large-scale prospective multicenter studies are warranted to validate the proposed threshold, confirm its predictive performance, and define its role in routine clinical practice.

## 6. Patents

No patents resulting from the work reported in this manuscript.

## Figures and Tables

**Table 1:** Baseline characteristics and intraoperative ventilatory parameters of the study population (n = 277).

Variable	All Patients (n = 277)
Male sex, n (%)	130 (46.9)
Age, years	67.4 (10.2)
BMI, kg/m^2^	25.8 (4.1)
ASA class, n (%)	
I	139 (50.2)
II	126 (45.5)
III	12 (4.3)
Pack-years	27.7 (31.7)
FEV1, % predicted	92.9 (20.6)
FEV1/FVC	0.73 (0.12)
DLCO, % predicted	73.4 (17.6)
COPD, n (%)	86 (31.1)
CAD, n (%)	65 (23.5)
CVD, n (%)	25 (9.1)
CKD, n (%)	23 (8.3)
CLD, n (%)	24 (8.7)
Diabetes, n (%)	50 (18.1)
Type of resection, n (%)	
Lobectomy	137 (49.5)
Bilobectomy	2 (0.7)
Segmentectomy	34 (12.3)
Wedge resection	104 (37.5)
Anatomic resection, n (%)	173 (62.5)
Vol-Decay, mL	10.8 (13.6)
Pres-Decay, cmH_2_O	0.7 (1.7)

Values are presented as mean (SD) for continuous variables and n (%) for categorical variables. Abbreviations: BMI, body mass index; ASA, American Society of Anesthesiologists; FEV1, forced expiratory volume in 1 s; FVC, forced vital capacity; DLCO, diffusing capacity of the lung for carbon monoxide; COPD, chronic obstructive pulmonary disease; CAD, coronary artery disease; CVD, cerebrovascular disease; CKD, chronic kidney disease; CLD, chronic liver disease.

**Table 2:** Univariate analysis comparing patients with postoperative prolonged air leak (PAL) and those without PAL.

Variable	Patients with PAL (n = 18)	Patients Without PAL (n = 259)	*p*-Value
Male sex, n (%)	6 (33.3)	128 (49.4)	0.19
Age, years	66.5 (11.1)	67.4 (10.2)	0.71
BMI, kg/m^2^	23.7 (20.7)	28.8 (24.9)	0.49
ASA class, n (%)			0.55
I	8 (44.4)	131 (50.6)	
II	9 (50.0)	117 (45.2)	
III	1 (5.6)	11 (4.2)	
Pack-years	43.2 (24.6)	26.5 (31.9)	0.04
FEV1, % predicted	80.3 (18.3)	93.8 (20.4)	0.007
FEV1/FVC	0.67 (0.14)	0.74 (0.12)	0.01
DLCO, % predicted	67.0 (12.4)	73.9 (17.8)	0.10
COPD, n (%)	7 (38.9)	79 (30.5)	0.55
Type of resection, n (%)			0.07
Lobectomy	10 (55.6)	127 (49.0)	
Bilobectomy	1 (5.6)	33 (12.7)	
Segmentectomy	1 (5.6)	1 (0.4)	
Wedge resection	6 (33.3)	98 (37.9)	
Anatomic resection, n (%)	12 (66.7)	161 (62.2)	0.70
Vol-Decay, mL	19.2 (25.3)	10.2 (12.2)	0.007
Pres-Decay, cmH_2_O	1.05 (1.6)	0.7 (1.7)	0.40

Values are presented as mean (SD) for continuous variables and n (%) for categorical variables. Abbreviations: BMI, body mass index; ASA, American Society of Anesthesiologists; FEV1, forced expiratory volume in 1 s; FVC, forced vital capacity; DLCO, diffusing capacity of the lung for carbon monoxide; COPD, chronic obstructive pulmonary disease.

## Data Availability

Not applicable.
